# Comparison of overexpansion capabilities and thrombogenicity at the side branch ostia after implantation of four different drug eluting stents

**DOI:** 10.1038/s41598-020-75836-6

**Published:** 2020-11-27

**Authors:** Pawel Gasior, Shengjie Lu, Chen Koon Jaryl Ng, Wee Yee Daniel Toong, En Hou Philip Wong, Nicolas Foin, Elvin Kedhi, Wojciech Wojakowski, Hui Ying Ang

**Affiliations:** 1 Division of Cardiology and Structural Heart Diseases, Medical University of Silesia, Katowice, Poland; 2 National Heart Research Institute Singapore, National Heart Centre Singapore, 5 Hospital Drive, Singapore, 169609 Singapore; 3 Department of Biomedical Engineering, National University of Singapore, Singapore, Singapore; 4 School of Material Science and Engineering, Nanyang Technological University, Singapore, Singapore; 5 Duke-NUS Medical School, Singapore, Singapore; 6 Hopital Erasme, Universtite Libre de Bruxelles, Bruxelles, Belgium

**Keywords:** Interventional cardiology, Cardiology

## Abstract

Interventions in bifurcation lesions often requires aggressive overexpansion of stent diameter in the setting of long tapering vessel segment. Overhanging struts in front of the side branch (SB) ostium are thought to act as a focal point for thrombi formation and consequently possible stent thrombosis. This study aimed to evaluate the overexpansion capabilities and thrombogenicity at the SB ostia after implantation of four latest generation drug-eluting stents (DES) in an in-vitro bifurcation model. Four clinically available modern DES were utilized: one bifurcation dedicated DES (Bioss LIM C) and three conventional DES (Ultimaster, Xience Sierra, Biomime). All devices were implanted in bifurcation models with proximal optimization ensuring expansion before perfusing with porcine blood. Optical coherence tomography (OCT), immunofluorescence (IF) and scanning electron microscope analysis were done to determine thrombogenicity and polymer coating integrity at the over-expanded part of the stents. Computational fluid dynamics (CFD) was performed to study the flow disruption. OCT (*p* = 0.113) and IF analysis (*p* = 0.007) demonstrated lowest thrombus area at SB ostia in bifurcation dedicated DES with favorable biomechanical properties compared to conventional DES. The bifurcated DES also resulted in reduced area of high shear rate and maximum shear rate in the CFD analysis. This study demonstrated numerical differences in terms of mechanical properties and acute thrombogenicity at SB ostia between tested devices.

## Introduction

Bifurcation lesions account for one-fifth of all coronary interventions and are predisposed to higher rates of in-stent restenosis, stent thrombosis, and recurrent adverse clinical events^1^. Although stent thrombosis may have a relatively low incidence (0.6–3.4%) it is still the most feared complication in coronary interventions due to the associated mortality risk for the patients^2^. The ideal management and treatment strategy for these complex lesions remain challenging and unclear^3,4^. The difficulty of PCI treatment for bifurcation lesions is presumably attributed to its high morphological complexity. Also, the size differences of the proximal vessel compared to the distal main and side branch (SB) can make it difficult for the stents to accommodate different diameters since most stents are optimized for a single size. It has been reported that about 23% of all stent thrombosis occur in bifurcation lesions^5^.

Provisional stenting (PS), i.e. stent in the main vessel eventually followed by SB intervention, represents the gold standard for unselected bifurcated lesions. Yet, one third of PS cases may require crossover to 2-stent approach and failures to deliver the second stent may occur^6–8^. Additionally, complex multiple stent strategies may result in considerable overlap and malapposition of the struts. It was reported that a two-stent strategy was associated with a stiffening process of the systolic and diastolic change in bifurcation angle, which was an independent predictor of adverse events^9^. Also, lesions located in bifurcations lesions are prone to delayed arterial healing following stent implantation^3^. This is especially important in the left main coronary artery (LMCA) bifurcation interventions where large amount of myocardium remains at risk and stenting techniques tend to be complex and are associated with high incidence of adverse events^10^. Therefore, provisional stenting seems to be preferred strategy for bifurcation lesion treatment, especially in non-severe side-branch ostial involvement. Bifurcation interventions often requires aggressive overexpansion of stent diameter in the setting of long tapering vessel segment. Also, overhanging struts in front of the SB ostium are thought to act as a focal point for thrombi formation and consequently possible stent thrombosis^11^. The latest generation drug eluting stents (DES) demonstrated improved vascular healing following implantation and are considered preferred stent choice in the bifurcation intervention^12^. As a consequence, the clinical outcomes after LMCA stenting have become comparable to coronary artery bypass grafting in the low- and intermediate-risk patients^13–15^.

Benchtop models and flow loop systems established by several groups demonstrated the benefits and reproducibility of this method to evaluate various stent platforms and techniques^16–20^. In bifurcation stenting, benchtop models and evaluations have been conducted to assess optimization of side branch re-crossing, crush, culotte and kissing balloon inflation techniques.

Data obtained from the bench testing can be further integrated with computational fluid dynamics (CFD) simulations to understand the local hemodynamic microenvironment in bifurcation stenting^21–24^. It is important to understand hemodynamic parameters as local disruption of physiological flow has been reported to modulate plaque progression and the degree of inflammation. Low endothelial shear stress (ESS) has been reported to increase plaque progression and a combination of both low and oscillatory ESS is considered to be particularly atherogenic^25^. High ESS, on the other hand, is a known risk factor for platelets activation. It has been demonstrated that in segments exposed to high shear rate, red blood cells cause platelets displacement toward the vessel wall via a process called margination. The activation and accumulation of platelets and adhesion proteins near the vessel wall results in a prothrombotic environment^26–28^. Results generated from 2D CFD models have demonstrated shear rate trends consistent with thrombogenicity trends observed experimentally^16,29,30^.

Hence, this study aimed to provide new data on the overexpansion capabilities on newer stent platforms in a bifurcation model. In this study we proposed to evaluate and compare the overexpansion capabilities and thrombogenicity at the SB ostia after implantation of bifurcation dedicated DES and three conventional latest generation DES in an in vitro bifurcation model. As the shear rate is an important hemodynamic parameter involved in thrombus formation, CFD will be used to supplement our understanding of thrombus formation in these bifurcation stenting.

## Methods

### Device description

Bifurcation dedicated DES (Bioss LIM C, Balton, Poland) and three conventional DES (Ultimaster, Terumo, Japan; Xience Sierra, Abbott, USA; Biomime, Meril Life Sciences, India) were used in this experiment. All tested devices are commercially available in CE mark countries. Detailed description of Bioss LIM C has been previously reported^31^. In brief, the Bioss LIM C platform is made of cobalt–chromium (Co–Cr) alloy with strut thickness of 70 μm. Bioss LIM C elutes sirolimus from the biodegradable coating consisting of a co-polymer of lactic and glycolic acids. The device is mounted on a rapid-exchange catheter with a semi-compliant stepped balloon. The Bioss LIM C stent consists of two main parts with different diameters. The ratio of the proximal part diameter to the distal one varies between 1.15 and 1.3, ensuring physiological compatibility and optimal flow conditions due to minimized flow disturbance as struts are apposed to the vessel wall. There is a 2.0–2.4 mm middle zone with two connecting struts. The Ultimaster coronary stent system consists of a Co–Cr platform featuring thin struts (80 µm for the 3.5 mm device) with a unique open-cell design for easy access to a SB and conformability to the vessel wall. The Ultimaster platform is coated with sirolimus in a matrix with bioresorbable, Poly(dl-lactide-co-caprolactone) polymer only on the abluminal surface. Xience Sierra is the newest generation of the extensively used XIENCE family everolimus-eluting stent. The device has Co–Cr platform with strut thickness of 81 µm. Everolimus is blended in a non-degradable polymer coated over another non-degradable polymer primer layer. The coating consists of acrylic and fluoro polymers. The Biomime DES features a Co–Cr platform and thin struts (61 µm) with a unique hybrid cell design comprising a mix of open and closed cells in order to provide optimal radial strength without compromising flexibility. The delivery balloon allows mediated stent expansion from middle (center) to edges during deployment. The Biomime platform is coated with sirolimus in a matrix of bioabsorbable polymers: poly-L-lactic acid and poly-lactic-co-glycolic acid. All stents were purchased from the respective manufacturers for the purpose of this study.

### Stent deployment

**Figure 1:**
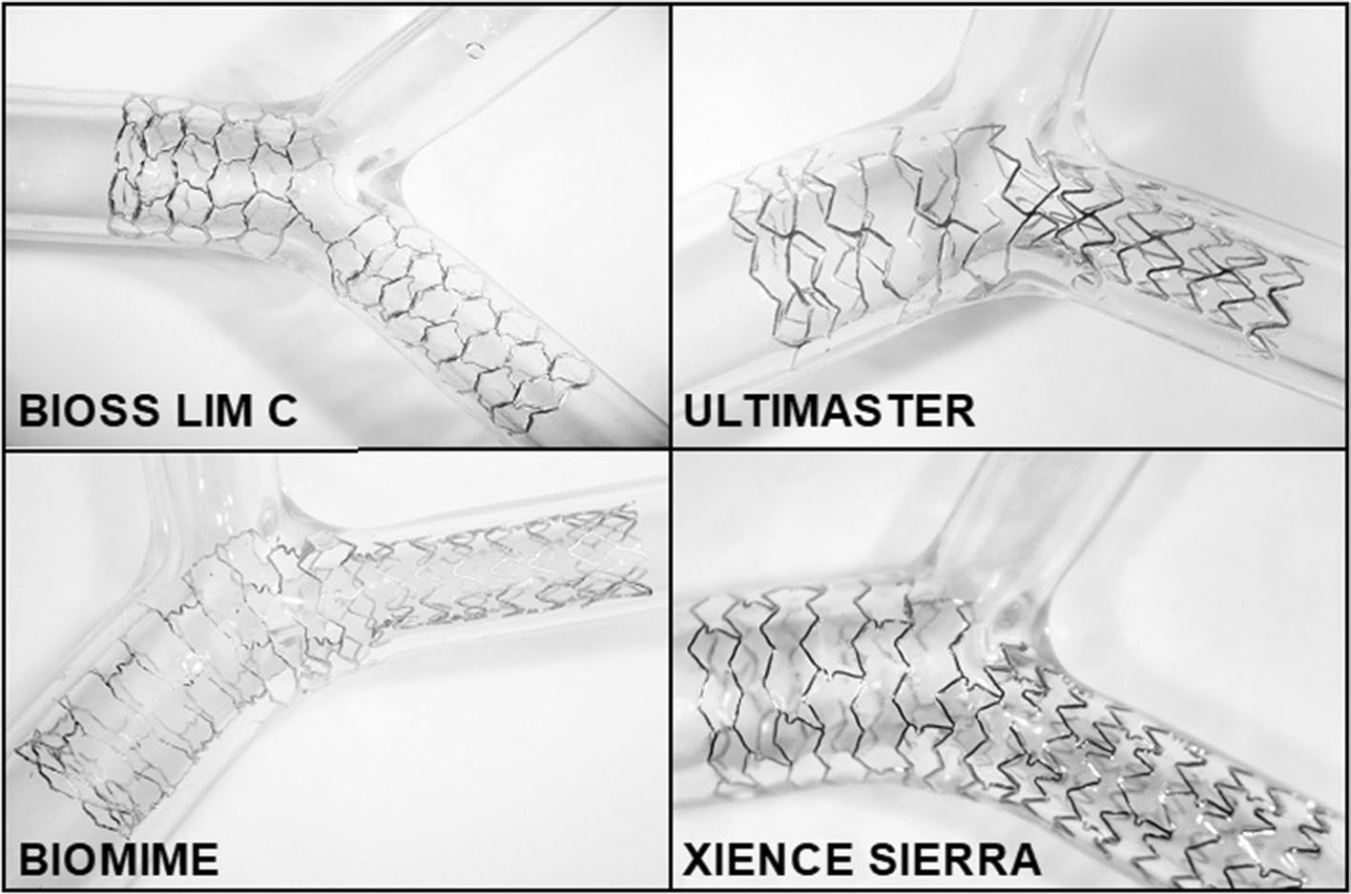
Optical images of stents deployed in bifurcation model.

The dimensions of the four DES employed in this experiment are described accordingly: Bioss Lim C (4.25–3.50 × 25 mm, n = 4, Balton, Poland), Ultimaster (3.5 × 24 mm, n = 4, Terumo, Japan), Biomime (3.5 × 24 mm, n = 4, Meril Life Sciences, India) and Xience Sierra (3.5 × 23 mm, n = 4, Abbott Vascular, United States). Stents were deployed in a Y-shaped left main bifurcation model (Inner Diameter: 5.5 mm, Main Branch Diameter: 3.5 mm, Side Branch Diameter: 3.5 mm, Material: Shore 40A Silicone with an angle of 90° between MB and SB). (Fig. 1) The inlet diameter was made larger than an average left main coronary artery diameter (4.5 ± 0.5 mm) to test the overexpansion capabilities of the stent platforms. Outlets were similar to the diameter of the left anterior descending coronary artery (3.6 ± 0.5 mm) and the left circumflex artery (3.4 ± 0.5 mm)^32^. The angle between the MB and SB was similar to the LAD-LCX angles measured clinically^9,33^. The angles between both branches and the bifurcation model were made to be the same at 135° after subtracting the angle between the MB and the SB at the bifurcation.

Diameters of the stent used were based on the diameter of the distal main branch (i.e. 3.5 mm). Stent were first deployed at nominal pressure (manufacturer stipulated inflation pressure value to achieve the intended diameter of the stent), with the distal half of the stent into the main branch (MB). Proximal optimization technique (POT) was done with a 5.5 mm percutaneous transluminal angioplasty dilatation catheter (Aviator Plus, Cordis) that was inflated to 14 atm (5.78 mm on the compliance chart) to ensure strut apposition to the vessel model wall in the proximal region. A post dilatation was done with a 4.0NC balloon (NC Quantum Apex, Boston Scientific) at 14 atm at the distal MB to ensure well apposition of stent strut in the distal MB.

**Figure 2:**
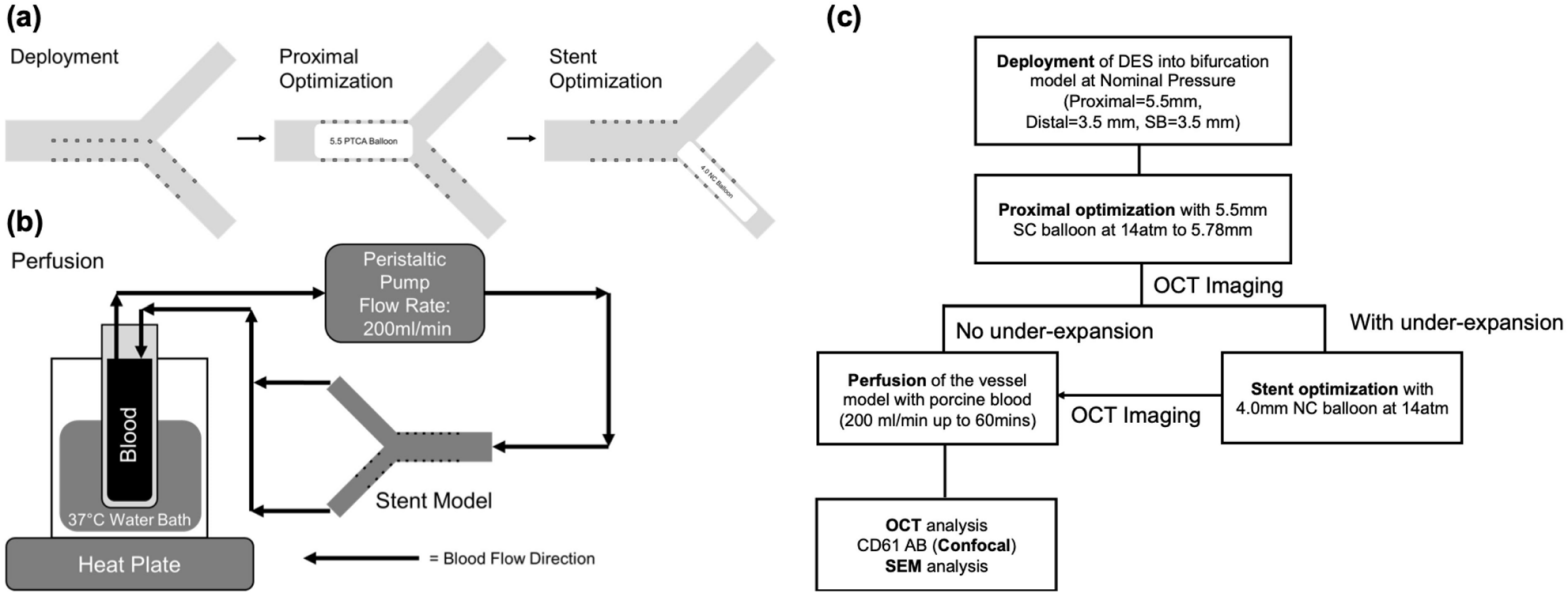
(**a**) Stent deployment process. (**b**) Schematic for flow perfusion setup. (**c**) Flow chart of experimental study.

Optical coherence tomography (OCT) pullbacks were obtained just before blood perfusion. A flow chart describing the study workflow is represented in Fig. 2c. OCT pullback was done at a pullback speed of 10.0 mm/s with a pullback length of 54.0 mm (540 frames) at a resolution of 15 µm.

### Flow perfusion

A peristaltic pump (Minipuls3, Gibson, United States) with fresh porcine blood and 10% anticoagulant (acid-citrate-dextrose) was used to perfuse the blood from a blood reservoir, through the stented models and back into the reservoir at a constant flow rate of 200 ml/min for 60 min (Fig. 2). This was described earlier and simulates the peak flow rate in a coronary artery^16,30^. Blood in the reservoir was heated with a heat plate to 37 °C to simulate physiological temperature during flow perfusion. After 60 min, perfused models were then flushed with 120 ml of Tyrode’s solution to remove excess blood before another OCT pullback was performed. After flow perfusion, the stents were removed carefully from the bifurcation model and subjected to immunofluorescence (IF) analysis and scanning electron microscopy (SEM) imaging. Additional care was taken to ensure that there is no loss and minimal contact with the clot on the stents during the removal process.

### Oct analysis

Using the OCT pullbacks before flow perfusion, the area, minimum, mean and maximum diameter of the proximal region of the stented model was measured every 2 mm. The elliptical index of the proximal region was also obtained as a ratio between the maximum and minimum diameter of each analyzed OCT frame^34^ (D_max_/D_min_ at 1 mm intervals, every 10 frames with a pullback speed of 10 mm/s) and normalized for the stent length. OCT cross sections were analyzed to quantify the thrombus area for each frame of the bifurcation region. Malapposed (MA) struts were defined as strut protruding into the lumen at a distance greater than the strut thickness^35^. Floating struts were defined as all the struts in the opening angle of the SB. The total number and percentage of well-apposed, malapposed and floating struts were counted for at the bifurcation ostium region. The thrombus area for each sample was calculated by selecting the three OCT frames with the largest measured thrombus area in the bifurcation region. The thrombus areas analysed from these three frames were averaged to obtain the thrombus area for that sample. OCT analysis was performed manually by a skilled operator.

### Immunofluorescence analysis

Immunofluorescence (IF) staining was done using the activated-platelet binding CD61 antibody (Bio-Rad, United States) for platelet aggregation quantification via IF analysis. Following an standard IF protocol from Leica Microsystems (Germany), the blood-perfused stents were sequentially incubated in paraformaldehyde, permeabilization (Triton X-100) and blocking buffers, which were all purchased from Thermo Fisher Scientific (United States). The samples were subsequently stained with CD61 antibody and secondary antibody. Confocal microscopy was then used to detect the IF over the whole stents at excitation of 485 nm. The longitudinal thrombus area (deemed as green IF) formed on at SBO of stent samples was measured by ImageJ software (NIH, United States).

### Drug coating integrity

After flow perfusion, the stents were removed carefully from the bifurcation model using two guide wires. Before removal of the stents, the vessel model was pressed gently at the distal and proximal ends so to facilitate the process without causing too much deformation to the main stent body. A tweezer was deployed at one end to remove the stent as gently as possible. All samples were subjected to the same removal protocol and the distal and proximal ends were not included in our analysis. After IF analysis, the stent samples were then imaged using SEM. The number of occurrences of strut coating damage spotted on the entire sample was then quantified and categorized into four different groups based on the degree of damage. The protocol was adapted from previously published study^36^. The different categories are:

- *Category 1*: Uneven coating, surface cracks or defects without bare metal exposure or webbing
- *Category 2*: Coating cracks or crater defects no greater than half the strut thickness in length or diameter with bare metal exposure.
- *Category 3*: Areas of coating lifting, coating cracks or crater defects greater than half the strut thickness but no greater than strut thickness in diameter with bare metal exposure.
- *Category 4*: Struts exhibiting coating dislodgement or crater defects greater than the strut thickness in diameter of the strut.

### Computational fluid dynamics

**Figure 3:**
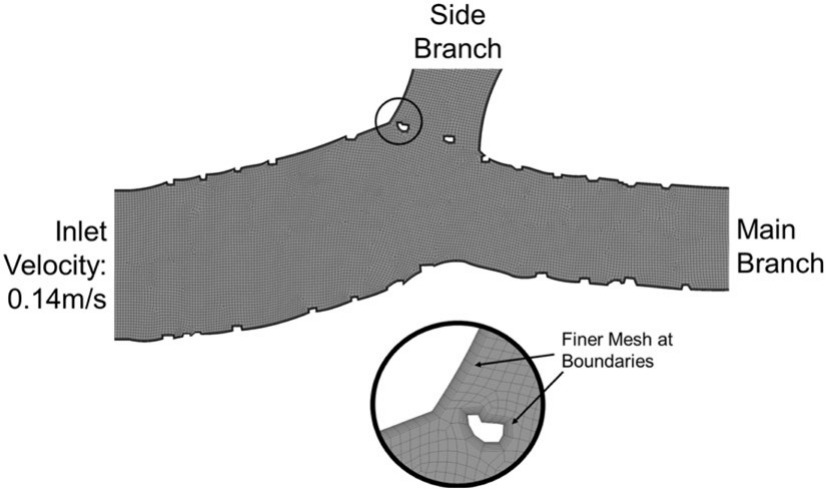
Representative meshed CFD model showing finer meshing along model boundary.

Flow patterns and shear rate were analyzed using computational fluid dynamics (CFD) to identify segments with higher risk of flow disturbance induced by malapposed and floating struts. 2D longitudinal geometries of the stented models were recreated using the OCT pullbacks. These geometries were then meshed and subsequently simulated with flow conditions similar to experimental conditions using a fluid computational software (Fluent, ANSYS). A quadrilateral dominant mesh was assigned to the models with a maximum element size of 0.1 mm, with finer mesh along the boundaries of the vessel wall as well as stent struts. (Fig. 3) A mesh convergence study was done to ensure that further refinement of element size will not greatly change the centre-line flow velocity of the model. The average number of elements per model was about 95,000. The model boundaries were modelled as rigid with no-slip condition. Blood was modelled as an incompressible, Newtonian fluid with a density of 1060 kg/m^3^ and a viscosity of 0.0035 Pa S^37^. A boundary condition of flow velocity of 0.14 m/s was implemented on the inlet of the model which is similar to the 200 ml/min flow rate in the experiment. The outlets were assigned as zero pressure outlets^38^. The area of high shear rate (> 1000 s^−1^) was obtained (physiological flow rate in normal human arteries falls within the range of 100–1000 s^−1^)^29^. In addition, maximum shear rate was obtained from these simulations.

### Statistical analysis

Statistical analysis was performed using SigmaStat statistical software (version 4.0; Systat Software, San Jose, California). Values are presented as median (interquartile range). Data were assumed to be nonparametric due to low number of samples and were analyzed with Kruskal–Wallis one-way ANOVA. If the ANOVA was significant, post-hoc testing was conducted comparing Bioss LIM C, Ultimaster, Biomime and Xience Sierra using Dunn's test. Differences were only considered significant when the calculated *p* value was < 0.05.

### IRB information

By regulation of the National Heart Research Institute Singapore, National Heart Centre Singapore, Singapore, no humans or animals were enrolled in the study, therefore, the approval of the local biological committee was not required. The fresh porcine blood was purchased from a local abattoir (Primary Industries Pte Ltd) and was approved by the Agri-Food and Veterinary Authority of Singapore.

## Results

### Optical coherence tomography and immunofluorescence

**Figure 4:**
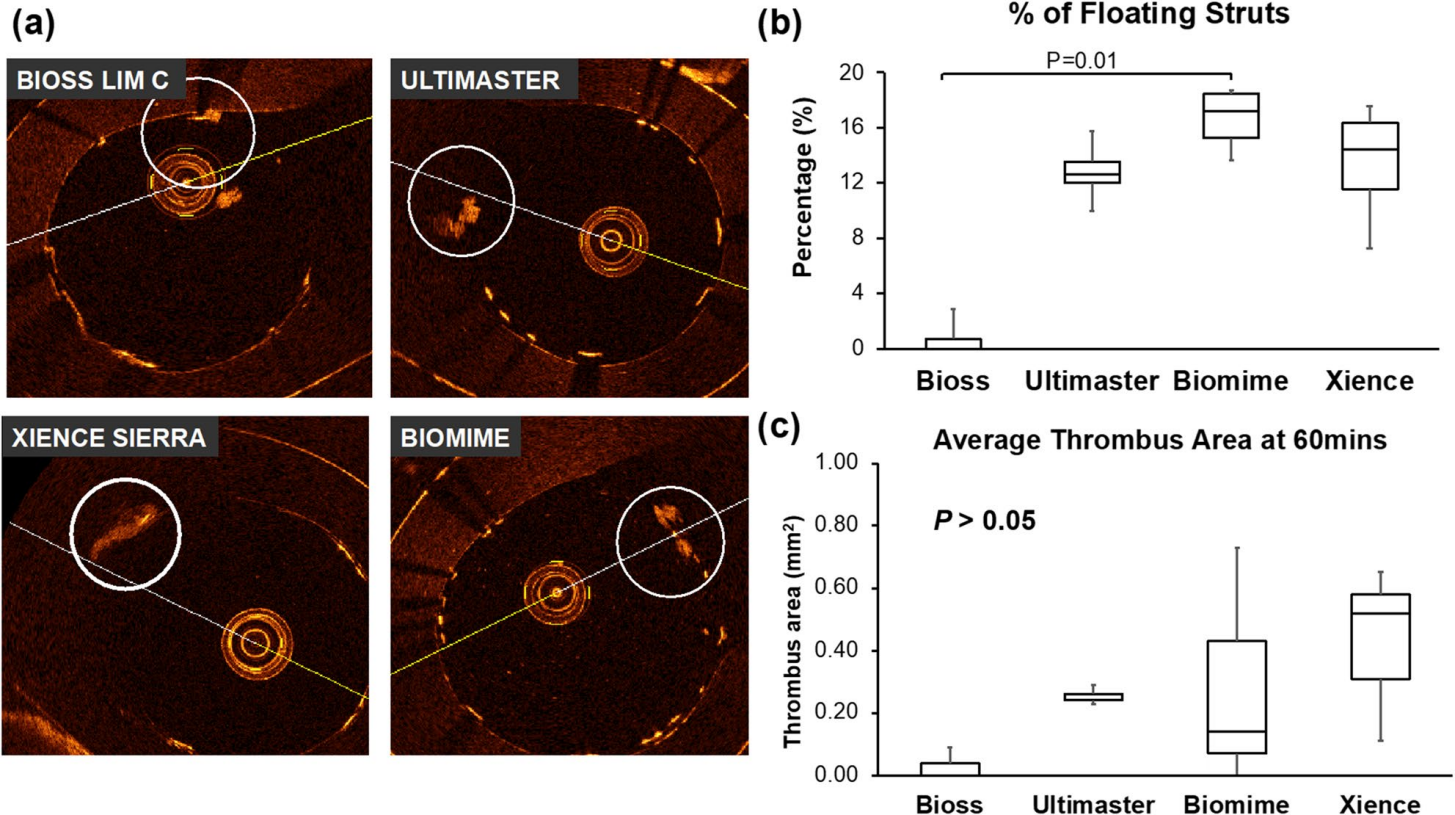
(**a**) Representative OCT images of thrombus formation on the stents at 60 min; (**b**) % floating struts and (**c**) average thrombus area based on OCT quantification.

A summary of OCT overexpansion and apposition data is presented in Table 1. Minimal, mean and maximal stent diameter were comparable between all devices. Also, stent area was similar in all tested groups. Eccentricity index (EI) was almost identical between all groups. The rate of well apposed struts was numerically higher in the Bioss LIM C group when compared to the conventional DES. The rate of floating struts was lowest in the Bioss LIM C with statistically significant difference between Bioss LIM C and the other three DES (*p* = 0.018). The rate of malapposed struts was numerically lowest in the Bioss LIM C. Thrombus area (based on OCT analysis) was numerically smaller in bifurcation dedicated DES when compared to the Ultimaster, Biomime and Xience Sierrra (Bioss LIM C: 0.00 (0.00–0.04) mm^2^ vs. Ultimaster: 0.24 (0.24–0.26) mm^2^ vs. Biomime 0.60 (0.37–0.67) mm^2^ vs. Xience Sierra 0.52 (0.31–0.58) mm^2^, *p* = 0.113). Representative OCT frames, % floating strut and thrombus quantification are presented in Fig. 4.

**Table 1:** Optical coherence tomography analysis of stents over-expansion.

	Bioss	Ultimaster	Biomime	Xience	*P* value
**OCT proximal diameter (mm)**					
Min	5.21 (5.18–5.28)	5.17 (5.11–5.24)	5.14 (5.09–5.22)	5.22 (5.22–5.32)	> 0.05
Mean	5.35 (5.30–5.44)	5.31 (5.27–5.35)	5.30 (5.25–5.34)	5.40 (5.37–5.49)	> 0.05
Max	5.48 (5.41–5.57)	5.47 (5.39–5.56)	5.48 (5.43–5.51)	5.56 (5.50–5.65)	> 0.05
**OCT proximal lumen area (mm**^**2**^**)**					
Area	22.51 (22.03–23.23)	22.15 (21.87–22.54)	22.05 (21.66–22.43)	22.89 (22.63–23.67)	> 0.05
**OCT proximal eccentricity index**					
EI	1.05 (1.04–1.06)	1.05 (1.04–1.06)	1.06 (1.05–1.07)	1.05 (1.04–1.06)	> 0.05
**OCT strut analysis**					
WA (%)	96.5 (92.8–99.1)	82.0 (79.8–82.6)	80.8 (80.6–81.0)	82.0 (77.3–86.0)	> 0.05
Floating (%)	0.0 (0.0–0.7)^a^	12.7 (12.0–13.6)	17.2 (15.3–18.5)	14.5 (11.6–16.4)	< 0.05
MA (%)	2.0 (0.9–5.0)	6.3 (4.6–8.4)	2.9 (0.6–5.8)	5.1 (4.0–6.4)	> 0.05

Values are presented as median (interquartile range).

*EI* elliptical index, *WA* wall apposed, *MA* malapposed.

^a^If *p* < 0.05 from Biomime.

**Figure 5:**
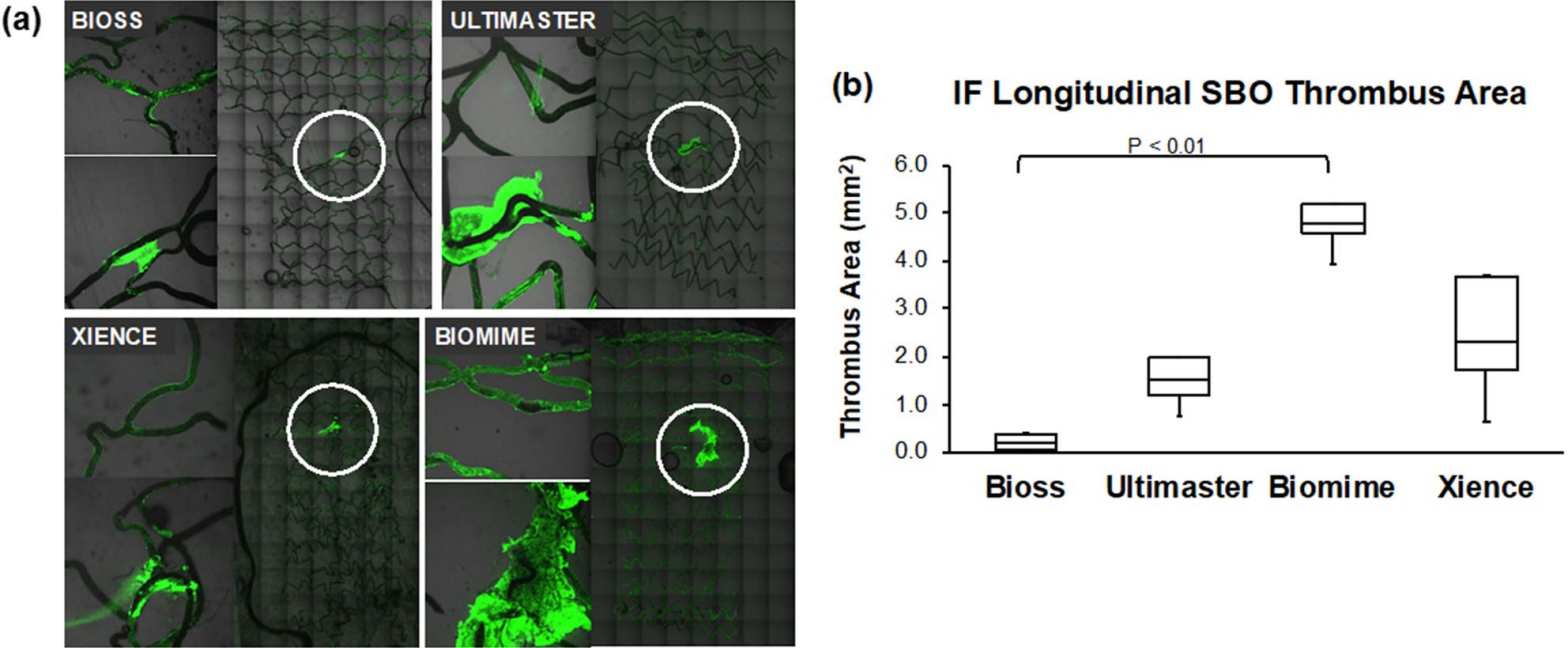
(**a**) Representative confocal images and (**b**) immunofluorescence (IF) quantification of thrombus formation on the stents at 60mins.

Immunofluorescence analysis demonstrated similar results as OCT analysis. Thrombus area obtained from confocal microscopy was also smallest in Bioss LIM C group with statistical significant difference between Bioss LIM C and Biomime stents (Bioss LIM C: 0.21 (0.07–0.39) mm^2^ vs. Biomime 4.80 (4.55–5.20) mm^2^, *p* = 0.007). Representative immunofluorescence images and longitudinal thrombus area are presented in Fig. 5.

### Computational fluid dynamics

**Figure 6:**
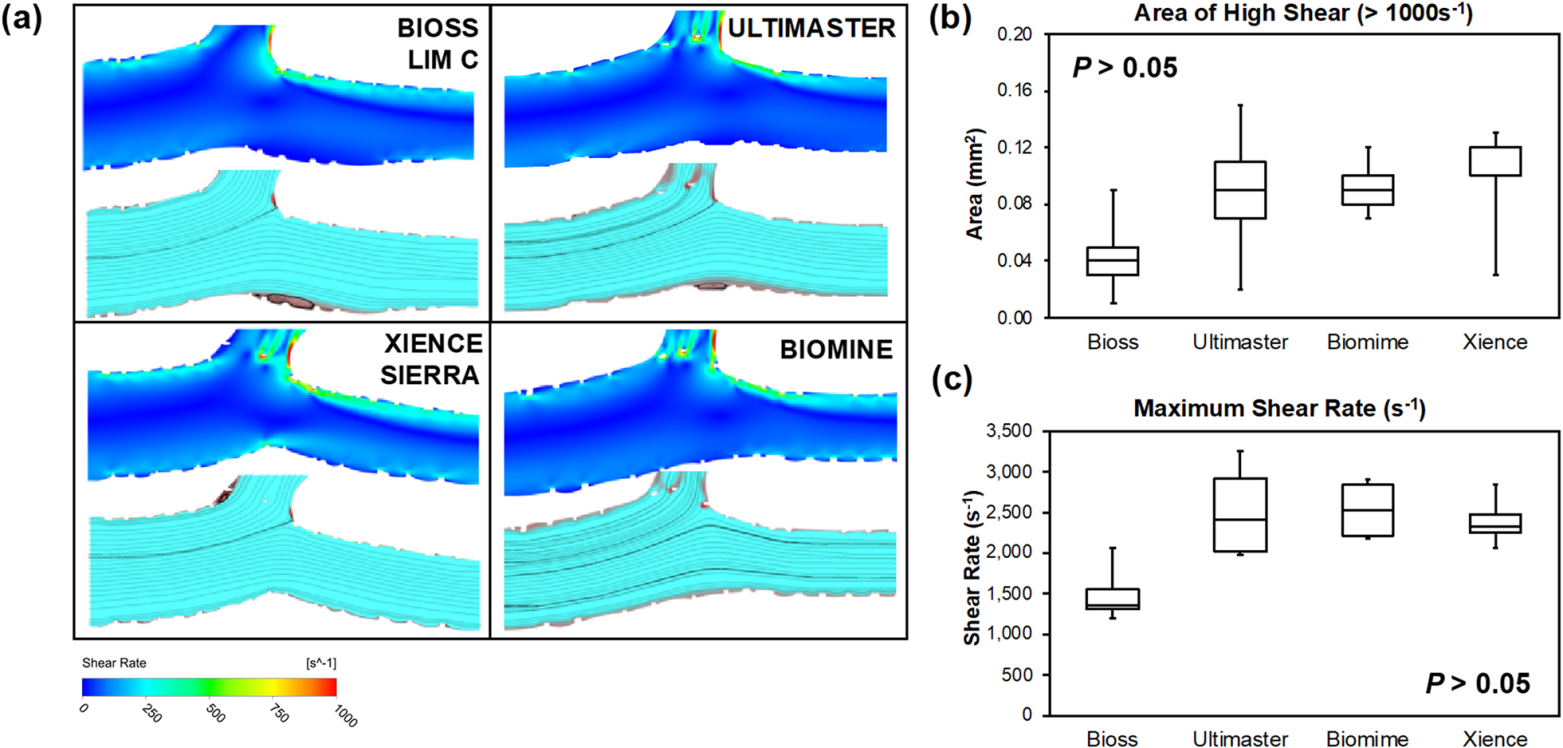
(**a**) Representative CFD images and (**b**) area of high shear (> 1000 s^−1^) and maximum shear rate quantification of the four stents.

Area of high shear rate (> 1000 s^−1^) was numerically lowest in the Bioss LIM C when compared to the conventional DES stents (Bioss LIM C: 0.04 (0.03–0.05) mm^2^ vs. Ultimaster: 0.09 (0.07–0.11) mm^2^ vs. Biomime: 0.09 (0.08–0.10) mm^2^ vs. Xience Sierra 0.12 (0.10–0.12) mm^2^, *p* = 0.353). Also, the maximum shear rate was numerically significantly lowest in the Bioss LIM C (Bioss LIM C: 1362 (1308–1548) s^−1^ vs. Ultimaster: 2413 (2015–2912) s^−1^ vs. Biomime 2528 (2221–2842) s^−1^ vs. Xience Sierra 2329 (2238–2483) s^−1^, *p* = 0.077). Representative images from CFD analysis, area of high rate and maximum shear rate quantification are presented in Fig. 6.

### Drug coating integrity

**Figure 7:**
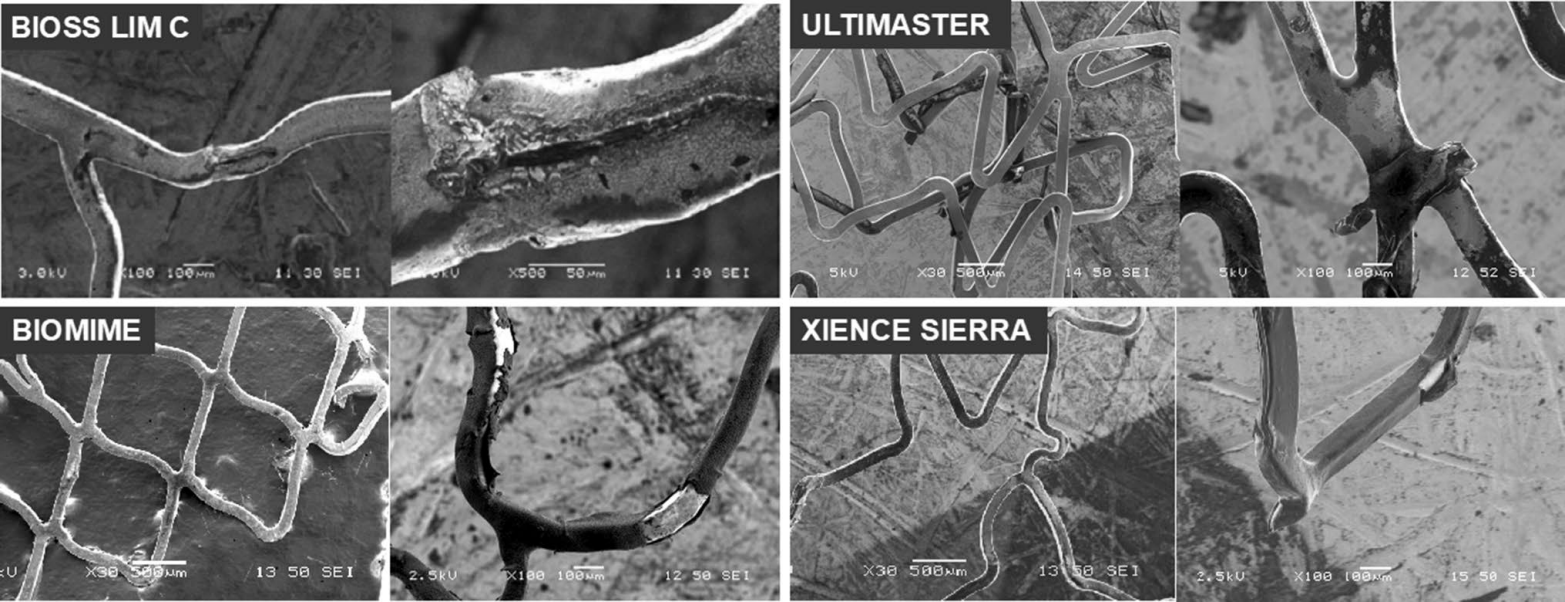
Representative SEM images of drug coating damage.

Summary of the SEM analysis of drug coating integrity is shown in Table 2. The highest number strut coating damage (in all categories) was observed in Biomime stent. Bioss LIM C displayed lowest number of coating damages from 2nd and 3rd category with the complete absence of the 4th category (Bioss LIM C: 0 vs. Ultimaster: 23.0 (18.8–34.3) vs. Biomime 33.0 (25.5–37.0) vs. Xience Sierra 9.0 (4.5–14.3), *p* = 0.007). Representative SEM images of drug coating damage are presented in Fig. 7.

**Table 2:** Scanning electron microscope analysis of polymer coating damage.

	Bioss	Ultimaster	Biomime	Xience	*P* value
**SEM coating analysis**					
Category 1	6.5 (5.5–13.5)	2.0 (1.5–6.8)	10.0 (7.8–13.8)	10.5 (9.3–15.0)	> 0.05
Category 2	0.5 (0.0–1.5)	0.0 (0.0–1.3)	7.0 (6.0–17.5)	7.0 (6.5–8.0)	< 0.05
Category 3	0.0 (0.0–0.3)^b^	1.0 (0.0–3.3)	8.5 (3.8–18.8)	3.5 (3.0–6.0)	< 0.05
Category 4	None^a,b^	23.0 (18.8–34.3)	33.0 (25.5–37.0)	9.0 (4.5–14.3)	< 0.05

Values are presented as median (interquartile range).

^a^If *p* < 0.05 from Ultimaster.

^b^If *p* < 0.05 from Biomime.

## Discussion

In this study, we compared overexpansion capabilities and acute thrombogenicity at the SB ostia after implantation of one bifurcation dedicated DES and three conventional DES in an in vitro bifurcation model. The major comparative findings of this study are that (1) bifurcation dedicated DES (Bioss LIM C) have significantly smaller thrombus formation at SB evaluated by OCT imaging and immunofluorescence analysis when compared to conventional DES, (2) reduced area of high shear rate and maximum shear rate in the CFD analysis; (3) lower incidence rate of drug coating damage in the over-expanded part of the stent.

Coronary artery bifurcation regions are prone to promote atherosclerotic plaque formations, and intervention in bifurcations especially in the LMCA setting carries higher risk of adverse events than elsewhere. Even in the modern DES era, the LMCA bifurcations are considered one of the most challenging lesion subsets. Implantation of a single stent in the main branch with provisional SB stenting was associated with favorable outcomes when compared to double stenting technique and currently is recognized as a technique of choice in the majority of bifurcation interventions^39–41^. Elective complex double stenting techniques were associated with worse clinical results when compared to the provisional approach^39,42^. Furthermore, even in the more challenging bifurcation anatomies intentional implantation of stents in both main branch and SB was not found to be beneficial^39^. However, careful selection of stent platforms in provisional stenting may have immense impact on the procedural outcomes and suitability of this technique for complex bifurcation anatomies. In-vitro bench testing can add to the anatomical and functional assessment of bifurcation lesions, guiding percutaneous therapeutic strategies. Insights from the bench tests studies provides important information that may be helpful for careful selection of stent size for contemporary DES based on model designs. Such information is especially critical in left main bifurcation stenosis treatment where overexpansion to larger, oversized diameter may be required to ensure full stent apposition. Given the strong connection between biological responses such as restenosis and thrombosis, and perturbations in wall shear stress, it is useful to compute shear stress. Previous published work on in vitro overexpansion of stents demonstrated correlation between benchtop data and CFD analysis. For analysis on stent thrombogenicity, Kolandaivelu et al. studied the impact of stent malapposition, as well as strut thickness, on acute stent thrombogenicity demonstrated that doubling the strut thickness increased thrombogenicity by 1.5-fold^16^. Another study conducted by Otsuka et al. used an ex vivo swine model and concluded that permanent polymer coating of Xience DES was significantly less thrombogenic than other biodegradable polymer-coated DES platforms^17^. However, most of these models used older generation of DES and there is insufficient studies on complex lesions such as bifurcation.

Overhanging struts in front of the SB ostium are thought to act as a focal point for thrombi formation and consequently possible stent thrombosis. Previous study using the same model demonstrated a direct causal effect between struts protruding into ostial site branch at bifurcation lesions and increased thrombogenicity^11,18,43,44^. Struts floating at the SB orifice were shown to affect thrombus formation mechanism. Our study demonstrated that thrombus area was lower in Bioss LIM C when compared to the conventional DES from OCT analysis. Immunofluorescence analysis concurred similar results with significantly smaller thrombus formation in bifurcation dedicated DES when compared to Ultimaster, Biomime and Xience Sierra with statistically significant difference between Bioss LIM C and Biomime. Lower thrombus formation in Bioss LIM C is most likely associated with the device architecture consisting of only two connecting struts at the SB ostia which reduces the metal-to-artery ratio, providing less luminal compromise at the level of the side branch in-flow. It is important to note that the larger initial dimension of the Bioss (4.25 mm vs. 3.5 mm) will result in Bioss not experiencing the same radial strain when expanded to 5.5 mm. This contributed to the lower amount of floating (0%) and malapposed struts (2%) seen in Bioss as compared to the other three stents.

Disruption of the laminar flow within coronary bifurcations promotes neointimal hyperplasia and plaque formation^45^. Even when the neointimal growth due to altered hemodynamics is inhibited by the elution of the antiproliferative agents from the DES surface, abnormal flow patterns can still lead to stent thrombosis^46^. There is paucity of data on flow alterations in bifurcation lesions associated with stent implantation. Constantly disturbed hemodynamic flow in the bifurcation regions might be associated with increased risk of restenosis and thrombosis. Regions at greater risk of thrombosis in the bifurcations commonly correspond to the regions with increased proliferative response and are located opposite to the flow divider. Previous study demonstrated that high wall shear stress regions are associated with plaque erosion and rupture and facilitate the accumulation of platelets and other blood thrombogenic factors close to the arterial wall^47^. In bifurcation lesions presence of struts at side branch in flow might alter blood flow resulting in increased high shear stress regions. In our study, area of high shear rate and maximum shear rate was numerically lower in the Bioss LIM C when compared to the conventional DES stents, which might also explain smaller the thrombus area in OCT imaging and immunofluorescence analysis. Again, the lesser alteration of blood flow might be attributed to the design of Bioss LIM C with only two connecting struts at the SB ostia.

Interventions in the LMCA bifurcation often requires aggressive overexpansion in the proximal segment of the stent. Application of the extreme forces during overexpansion on the stent struts increases the risk of polymer coating damage and strut deformation^48^. Bench test studies demonstrated that contemporary DES platforms are able to expand largely beyond their nominal diameter^49^. Similarly, in our study all platforms were able to expand well above nominal diameter without any signs of strut fracture or deformation. However, it should be emphasized that despite the fact that most of contemporary DESs can be largely oversized does not imply that excessive post-dilatation is safe. Approaching the physical limit of the stent might be associated with alteration of device performance by affecting their mechanical stiffness and drug coating integrity^49^.

Furthermore, drug coating damages or detachment of debris might be associated with increased risk of thrombosis and inflammation with neointimal reactions^2,50^. The lowest number of strut coating damage (in all categories) was observed in Bioss LIM C. This favorable result might be attributed to the design of the Bioss LIM C with larger diameter of the device in the proximal part (by 0.75 mm) when compared to conventional DES which ensures lower overexpansion of the device and subsequently smaller strut coating damage. This might also explain the higher rate of well apposed struts in bifurcation dedicated DES assessed by OCT imaging when compared to conventional DES. Both Bioss LIM C and Biomime maximal expansion diameter allowed by the manufacturer is smaller than overexpansion achieved by post-dilatation in our study. While Xience Sierra and Ultimaster are stated by the manufacturers to have 5.5 mm overexpansion limit for the size used here.

## Limitations

Measurements obtained from this study should be carefully interpreted as the stents were deployed in vitro in static conditions without the presence of a constraining lesion to limit the expansion of the stent. Therefore, this benchtop model cannot predict what occurs in patients at clinical setting. It is important to note that this study compared stents implantation in the bifurcation with a provisional approach and without any SB optimization, thus the results only apply to this case. In addition, deployment of the BIOSS stent in this study was idealized to ensure that the connectors do not disrupt flow into the SB. This might not be as reproducible clinically and hence the results may differ in real practice. Also, the simplified model of left main bifurcation anatomy does not fully reflect the arterial tissue response to stent deployment physiologically or the complexity of the other bifurcation anatomies. Hence, the results obtain are only an approximation of the actual in vivo behavior of the stent-artery response during overstretching^51^. Furthermore, the in-vitro bench model used in this is study made of linearly elastic material in contrast to hyper-elastic arterial tissue. This results in different deformation of both stents and in-vitro bench model when compared to stent deployment in human arteries. Perfusion of the stented model was at a steady state, rather than the pulsatile nature of flow physiologically, which might affect the results of our studies. However, existing literature has shown that the use of steady flow was acceptable as thrombus formation is more affected by the shear generated than flow pulsatility^52^.

This in vitro bench top experiments represents acute thrombogenicity of stent deployment^16,18^, and further testing in animal model will be required to fully understand the long-term effects of these stents on stent thrombogenicity. For the drug coating integrity experiment, the purpose of this study is to compare between bifurcation dedicated DES and DES, hence the Ultimaster and Biomime stent designs used here are not the latest generation of devices (e.g. Ultimaster Tansei and Biomime Branch) which are more suited for over-expansion/bifurcation. Also, some of the strut coating damage might be related to retrieval of the devices from the silicone model. Although precaution was taken during the retrieval of devices from the silicone model, there might still be some loss of thrombus during the process and IF analysis.

2D models are limited in their ability to capture flow disruption in three direction and are unable to fully capture differences between models with different stent design. However, previous studies using 2D models have demonstrated shear rate trends consistent with experimentally observed thrombogenicity trends^16,23,29^. Future improvement can include building 3D CFD models which can recreate patient specific coronary anatomy^53^ and complex strut configurations, even for models with more than one stent^54^, for more accurate hemodynamic results. This can be achieved by utilizing additional imaging modalities like an angiogram or an X-ray in combination with OCT in order to capture the curvature of the vessel models accurately.

## Conclusions

Our study demonstrated that In vitro bench testing is a useful tool adding to the anatomical and functional assessment of bifurcation lesions, which allows detailed evaluation of tested devices and might led to improvement of DES technologies. This model suggested numerical differences in terms of mechanical properties and thrombogenicity at SB ostia between tested devices. Therefore, the model used in this study provides comprehensive in-vitro evaluation of the stent performance in coronary bifurcation setting and, in the future, might be applied to more devices and implantation techniques as well as varied bifurcation geometries. Furthermore, the bifurcation dedicated Bioss LIM C showed lower acute thrombogenicity when compared to conventional DES in our benchtop model. These findings might be potentially relevant as the enrollment of the prospective, multi-center single study in patients with an indication for distal unprotected left main revascularization has already started^55^.
